# Futures Challenges in Thyroid Hormone Signaling Research

**DOI:** 10.3389/fendo.2016.00058

**Published:** 2016-06-22

**Authors:** Frédéric Flamant

**Affiliations:** ^1^École Normale Supérieure de Lyon, CNRS, INRA, Institut de Génomique Fonctionnelle de Lyon, Université Claude Bernard Lyon 1, Université de Lyon, Lyon, France

**Keywords:** thyroid hormones, nuclear receptor, ChIPseq, signal transduction, transcription, genetic

## Abstract

The canonical pathway of thyroid hormone signaling involves its binding to nuclear receptors (TRs) acting directly on the transcription of a number of genes. Recent genome-wide studies revealed that chromatin occupancy by TR is not sufficient for transactivation of gene expression. Reciprocally, in some cases, DNA binding by TR may not be required for cellular response. This leaves many new questions to be addressed in future research.

## A Canonical Pathway for Thyroid Hormone Signaling

In 1986, the genes that encode the TRα1 and TRβ1 nuclear receptors of thyroid hormone (triiodothyronine or T3), now called THRA and THRB, were cloned (1–3). This breakthrough started years of intense investigations (4), which established what we will call here the canonical T3 signaling pathway. The key observations were:

– THRA and THRB belong to a superfamily of 48 genes present in the mammalian genome, which also encode nuclear receptors. The mode of action of these nuclear receptors shares a number of common features. Like many other nuclear receptors, T3 receptors (TRs including TRα1, TRβ1, and TRβ2 an alternate product of THRB later found in few cell types) act as ligand-dependent transcription factors.
– The structure of TRs is modular: the N-terminal domain binds DNA and is linked by a “hinge” to the C-terminal domain, which binds T3 and is required for heterodimers formation. TRα1, TRβ1, and TRβ2 display extensive structural similarities, with the exception of an N-terminal extension, which is absent in TRα1.
– Like a subset of other nuclear receptors, TRs act mainly as heterodimers with the RXR nuclear receptor. RXR/TR heterodimers can bind to DNA elements made of two half-sites, related to the 5′-AGGTCA-3′ consensus, organized in tandem and separated by four nucleotides (DR4).
– DNA binding is not ligand dependent. Unliganded TRs recruit transcription corepressors and exert a negative influence on gene expression. T3 binding changes the conformation of the ligand-binding domain of TRs. According to the “mouse trap” model, the repositioning the C-terminal helix 12 favors the recruitment of transcription coactivators at the expense of transcription corepressors. Both types of cofactors leave long-lasting marks on histone tails, which eventually modifies the recruitment of type II polymerase and the transcription initiation rate or neighboring genes.

Although many progresses have been performed during the following 20 years, this canonical model still holds true. It is sufficient to explain most of the pleiotropic influence of T3 in physiology and development, and in the corresponding pathologies. However, a number of problems remain to be solved, which are highlighted below. The choice is personal, and the goal is to stimulate new investigations.

## The Allosteric Properties of the TR/RXR/DR4/Coactivator Complexes and Their Pharmacological Consequences

Studies of other nuclear receptors indicate that DNA-bound nuclear receptor dimers have allosteric properties, meaning that ligand binding, DNA binding, heterodimerization, and cofactor recruitment are interdependent processes and that local changes in receptor structure can influence distant domains of the protein. A systematic study of the estrogen receptors confirm that the DNA sequence of the response element, the type of receptor, the ligand, and involved coactivators all contribute to define the transactivation capacity of the complex in a highly unpredictable manner (5, 6). Structural analysis of the glucocorticoid receptor more precisely analyzed how changes in the nucleotide sequence of the hormone-response DNA element have broad consequences on dimer conformations, DNA-binding kinetics, and transcriptional activity (7). Similarly, a single amino-acid substitution was found to deeply modify the repertoire of target genes (8). Structural analyses suggest that such long-distance allosteric effects also take place within TRs (9). For example, mutations in the C-terminal helix, located far from the ligand-binding pocket can reduce the affinity for T3 binding (10). Allostery also explains why some RXR-containing heterodimers are “permissive” for RXR, meaning that they can be activated by RXR agonists, while others are not (11, 12). RXR/TR heterodimers are thought to be always non-permissive for RXR, but one recent study suggests that there may be exceptions, in specific cellular contexts (13, 14). Therefore, a number of parameters, which are often overlooked, interplay to decide whether the recruitment of polymerase II and the transactivation of the neighboring gene will occur: exact sequence of the DNA-binding site and neighboring nucleotides, TR isotype, RXR isotype, and ligand.

This conclusion is important for the development of new TR ligands. Such synthetic compounds have been synthesized to develop thyromimetic drugs, which are expected to provide the beneficial effects of T3, notably cholesterol lowering, without having its cardiac toxicity. In addition to pharmacodynamics parameters, which, for example, tend to favor accumulation in hepatocytes, the allosteric influence of ligand binding may have important consequences on the receptor function (9). This has been elegantly exploited to synthesize a molecule able to activate an otherwise inactive mutant TRβ1 (15). Allosteric properties of TR/RXR/DR4 complexes might also be relevant for studies of thyroid hormone disruptors, as some of these environmental chemicals are thought to be TR ligands (16). Allostery also raises the intriguing possibility that thyroxine (T4), usually only considered as an inactive precursor of T3, and other natural iodinated compounds with different affinities for TRs (17), could trigger a distinct cellular response. Understanding allosteric properties of complexes would benefit from the resolution of the complete structure of the DNA-bound heterodimers, a very difficult task, which has only been achieved for PPAR/RXR, LXR/RXR, and VDR/RXR for the moment.

## Genome-Wide Studies Open a Wide Field of Investigation

The advent of deep DNA sequencing allowed transposing a number of molecular biology assays at genome-wide scale. This deluge of datasets is not only broadening our view but also questioning textbook knowledge on gene expression. For example, the distinction between promoters and enhancers is now uncertain (18). It also appears that enhancers are transcribed on both strands, as is a large fraction of the genome (19). In several cell types, it is now visible that transcription initiation is not necessarily the rate-limiting step of gene expression, due to the presence of a poised polymerase II (20). All these novelties have not yet been integrated in the molecular models of nuclear receptors function and will certainly influence our view on their mode of action.

In the field of T3 signaling, RNAseq, replacing microarray analysis, already produced a number of heat maps and lists of genes, from which it is difficult to extract a unifying picture for the moment (21). Taken together, these data confirm that different cell types respond to T3 in very different manners. Whether the gene induction is directly mediated by TR, or rather secondary to the rapid induction of another gene encoding an intermediate transcription factor, cannot be easily inferred from transcriptome analyses. Using an inhibitor of mRNA translation indicates that such secondary response starts within few hours after T3 stimulation and that only a minority of the reported T3-responsive genes are directly regulated by TRs (22). Chromatin occupancy can be assayed at genome-wide scale (ChIPseq) to identify all TR-binding sites (the TR “cistrome”) present next to TR responsive genes and better identify TR target genes. Three such studies mapped the sequence responsible for the T3 response of neural cells and hepatocytes (23–25). Two of these studies relied on tagging the N-terminus of TRs, to circumvent the difficulty of using antibodies raised against TR, which were thought to be of insufficient quality. However, the extensive overlap between the results later obtained with a commercially available antibody is reinsuring. This is clearly just a beginning, but these ChIPseq data brought a number of new questions.

ChIPseq analyses indicate that DR4 elements are predominant over other types of T3 response elements. While DR4-like elements are very frequent in the genome (>70 000), only a small subset is occupied in a given cell type. For other nuclear receptors, this type of situation is thought to result from the existence of “pioneer” factors required to open closed chromatin before nuclear receptor binding (26). However, there is no indication that such pioneer factors exist for TR binding. Furthermore, TRs seem able to create localized “open chromatin” structures after ligand binding (24). The significant overlap between the cistromes of hepatocytes and neural cells is in line with the possibility that chromatin compaction is not the determinant for DR4 choice. This overlap contrasts with striking differences in chromatin opening in these cell types, as judged by DNaseI hypersensitivity (21). The occupancy of only a small fraction of the DR4 elements present in the genome is thus unexplained for the moment.

Another surprise is the limited correlation that is observed between the occupation of a DR4 by TR and the transactivation of the neighboring gene after T3 treatment. Also, more than half of the rapidly responding genes possess a TR-occupied site within 20 kb of the transcription start site, TR binding is only observed at very long distance (>50 kb) for a number of T3-responsive genes. Such long-distance regulation could be explained by chromatin looping, which brings distant sequences at vicinity (27). Reciprocally, and more surprisingly, a number of expressed genes with a proximal DR4 occupied by TR do not respond to T3. This fact is not understood, and outlines the need to better understand genome compartmentation, as insulating structures could define the distance at which TR can transactivate. Again, the possibility exists that the allosteric properties of the TR/RXR/DR4 complexes define their transactivation capacity. This could explain why, in the same cells, genes can display a marked preference for TRα1 or TRβ1, as this preference is not correlated with differential chromatin binding of the receptors (23).

Genome-wide scale data also indicate a very significant overlap between the cistrome of different nuclear receptors within the same cell type. ChIPseq data in mouse liver are available not only for TR but also for several other nuclear receptors, including LXR, PPARα, PXR, and RXR (28, 29). As the physiological functions of the respective ligands of these receptors are clearly different, the extensive overlap between the cistromes is surprising. However, LXR/RXR, PXR/RXR, RXR/RXR, and RXR/TR dimers can all bind *in vitro* to DR4 elements and apparently recruit the same types of transcription cofactors. In this respect, it is also puzzling that the overlap is not complete for these receptors. This situation entails that a number of genes should be equally activated by these receptors and that these nuclear receptors should cross-talk. TRβ1 does not leave any “footprint” on DNA (24) confirming a very unstable association with DNA and transient occupancy (30). If the binding of the other heterodimers is also labile, one should expect that the interaction between the pathways will not be competitive, but additive or synergetic. Furthermore, the existence of composite elements with more than two half-sites and the presence in nuclei of very large protein complexes containing several nuclear receptors (31) suggest a number of additional possibilities for cross-talks with the other nuclear receptors, which do not bind on DR4. Few examples have been already studied (32).

Transcriptome analyses in liver and brain cells indicate that genes can be either up- or downregulated after T3 stimulation. In pituitary-derived cells, in which the direct negative influence of TRβ1 on *Thsb* gene promoter is well documented, there is strong evidence that TRβ1 can be recruited to chromatin and bind *Thsb* gene regulatory sequences (33). Therefore, it seems that some unknown molecular mechanism can turn liganded TRβ1 into a transcription repressor. What is the underlying molecular mechanism for T3-mediated transcriptional repression is currently unclear. Assuming that the mechanism is not peculiar to pituitary thyrotropic cells and might involve an unusual association of TRβ1 to chromatin at specific loci, ChIPseq data were thus eagerly expected. A global analysis revealed, however, that, at least in hepatocytes and neural cells, the genes which expression is negatively regulated after T3 stimulation do not usually possess a proximal-binding site occupied by TR. The most likely explanation is that the observed decrease in mRNA level, which becomes measurable only several hours after T3 treatment, is an indirect consequence of a rapid response to T3, for example, the induction of a gene encoding a transcription repressor. This is, however, a temporary conclusion, as a more direct analysis of transcription initiation has not been performed. Analyses of nascent RNA and of chromatin occupancy by RNA polymerase II are feasible at genome-wide scale and would bring a direct view of the transcriptional machinery.

## Going Beyond the Canonical Pathway

An abundant and controversial literature reports that T3 stimulates the generation of second messengers such as Ca^2+^, NO, inositol trisphosphate, and cAMP in various cells, in a rapid manner, which is incompatible with a transcriptional response (34). The changes influence the Akt and PKC signaling pathways, which eventually lead to a modification of gene expression (35). These rapid pathways are called “non-genomic,” although this designation can be confusing, because on the long term, they must eventually influence genome expression. Furthermore, as neurons without TR do not display any residual transcriptional response to T3 (22), the non-genomic response, at least in this cell population, probably involves proteins encoded by THRA and/or THRB. This also suggests that the genomic and non-genomic responses are not entirely independent pathways.

Three of the proposed pathways for non-genomic signaling are compatible with an intervention of THRA- and THRB-encoded proteins. Two of them involve the 30 and 43-kDa proteins encoded by THRA, both produced by alternate translation of TRα1 mRNA initiation in a number of cell types. The 30-kDa, corresponding to the isolated ligand-binding domain, has been found to be at the plasma membrane, where it stimulates Akt pathway (36). This is discordant with an older study claiming that the full-length TRα1 was activating this pathway in endothelial cells (37). By contrast, the 43-kDa protein has been detected by Western blotting in purified mitochondria (38). It still possesses the ability to bind DNA and has been proposed to act as a regulator of mitochondrial genome expression (39). Importantly, transgenic mice with selective elimination or overproduction of the 43-kDa protein have been produced, with significant developmental and physiological consequences (40, 41). Another study (42) concluded that a fraction of the full-length TRβ1 is also present at the plasma membrane. Unlike TRα1, which lacks a critical tyrosine residue, TRβ1 can serve as an intermediate between tyrosine kinase receptors and the PI3K/Akt pathway. Here again, this claim is supported by genetic evidence in mice (42, 43). Although self-consistent, all these studies lead to conclusions that are clearly not compatible, and it is likely that some of the proposed mechanisms will not resist deeper investigations. Settling this controversy would be easier if only few relevant cellular models were chosen and if the authors could demonstrate the presence of THRA- or THRB-encoded proteins without using artificial overexpression. This last point is critical, and technically challenging, as the proteins abundance is generally very low, even in the nucleus. The molecular details explaining the subcellular localization and activity of all these proteins and others THRA products that are not mentioned here (44) remain in any case to be understood. Future investigations should also clarify how and why vertebrate evolution generated such a complexity.

## Conclusion

Despite its well-recognized weaknesses, the canonical model resisted well over the last decades. Recent technological progresses bring the promise that we will soon reach a deeper and broader understanding of thyroid hormone mode of action. As the hormone fulfills many different functions, this would have important consequences in a number of different scientific fields.

## Author Contributions

The author confirms being the sole contributor of this work and approved it for publication.

## Conflict of Interest Statement

The author declares that the research was conducted in the absence of any commercial or financial relationships that could be construed as a potential conflict of interest.
